# DLK1: a novel therapeutic target in cancer

**DOI:** 10.1530/ERC-25-0513

**Published:** 2026-05-21

**Authors:** James Frederick Henry Pittaway

**Affiliations:** Centre for Endocrinology, William Harvey Research Institute, Barts and The London School of Medicine and Dentistry, Queen Mary University of London, London, UK

**Keywords:** DLK1, cancer, therapy, treatment, target

## Abstract

**Graphical Abstract:**

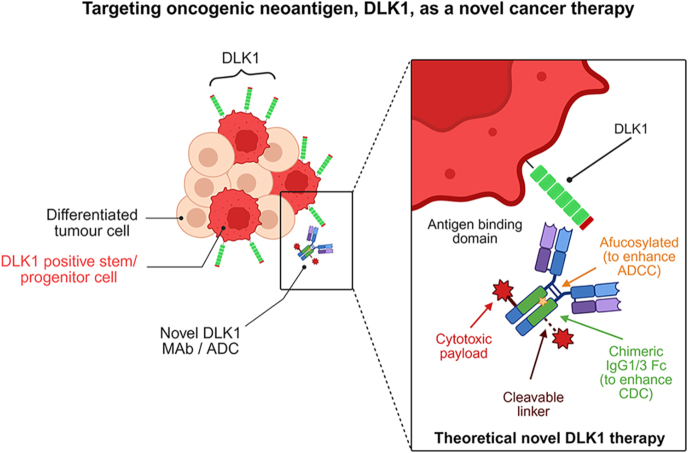

**Abstract:**

Delta-like non-canonical Notch ligand 1 (DLK1) is a cleavable transmembrane protein with tightly regulated, developmentally restricted expression. It is highly expressed during embryogenesis, where it plays a key role in controlling cellular differentiation and proliferation, but is largely silenced in adult tissues, persisting mainly within stem and progenitor compartments of endocrine organs. Notably, DLK1 is consistently re-expressed across a broad range of malignancies, with the highest prevalence observed in endocrine and neuroendocrine tumours, including adrenocortical carcinoma, phaeochromocytoma/paraganglioma, medullary thyroid carcinoma, and neuroblastoma. DLK1 expression is associated with adverse clinical outcomes and is increasingly implicated in maintaining a de-differentiated, stem-like tumour phenotype that might contribute to tumour progression and therapeutic resistance. The restricted expression of DLK1 in normal adult tissues, combined with its cell-surface localisation and functional relevance in tumour biology, makes it an attractive therapeutic target, particularly in endocrine malignancies where targetable options remain limited. Multiple DLK1-directed strategies are now advancing through preclinical and early clinical development, including afucosylated monoclonal antibodies, antibody–drug conjugates, dendritic cell vaccines, chimeric antigen receptor T-cell therapies, and radioimmunotherapy. Early-phase studies demonstrate encouraging safety profiles and signals of efficacy, with emerging evidence suggesting that tumour-specific factors – such as steroidogenesis, immune microenvironment, and drug efflux mechanisms – may influence response in endocrine cancers. This review collates current evidence on DLK1 biology and therapeutic targeting, with a focus on endocrine and neuroendocrine malignancies. We highlight key novel mechanistic insights, translational challenges, and future opportunities to exploit DLK1 as a precision therapeutic target in these high-need cancer subtypes.

## Introduction

**Figure 1:**
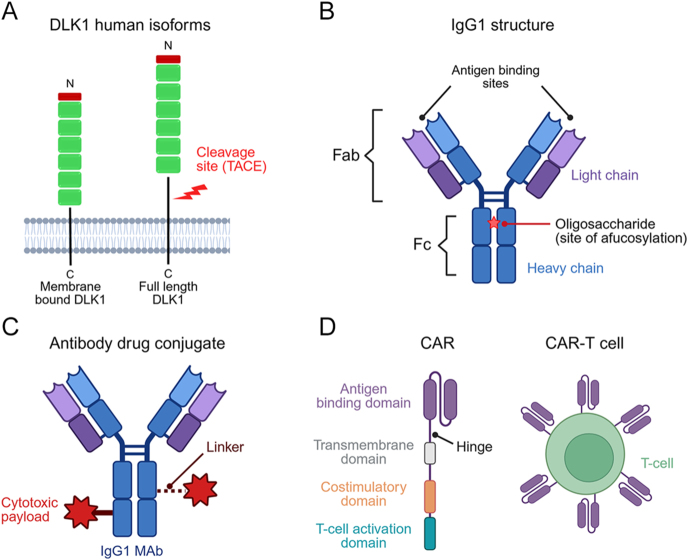
Diagrams of DLK1, MAb, ADC, and CAR-T. (A) DLK1 is a transmembrane protein that exists as two isoforms in humans, differentiated by the presence of an enzymatic cleavage site under the influence of TNFα-converting enzyme (TACE). (B) Structure of IgG1 – the most common monoclonal antibody (MAb). (C) Diagram of an antibody drug conjugate (ADC). (D) Structure of a chimeric antigen receptor (CAR) and its expression in a T-cell (CAR-T). Created with Biorender.

Delta-like non-canonical Notch ligand 1 (DLK1) – previously reported as *dlk*, fetal antigen 1 (FA1), pre-adipocyte factor 1 (Pref-1), and *pG2* – is a single-pass, transmembrane glycoprotein. It consists of six epidermal growth factor (EGF)-like repeats, a transmembrane domain, and a small intracellular tail. In humans, there are two isoforms, one full-length and one membrane-bound, differentiated by the presence of an enzymatic cleavage site at the juxtamembrane region in the former. The active ligand is cleaved from the full-length isoform under the action of TNFα-converting enzyme (TACE) (Fig. 1A) (1, 2).

The *DLK1* gene is located on chromosome 14q32 in humans and 12qF1 in mice (3, 4). It is paternally expressed from the imprinted *DLK1-DIO3* gene cluster. Epigenetic regulation at this locus, through methylation of imprinting control regions, is responsible for expression of both DLK1 and the largest cluster of non-coding RNAs in the human genome, including numerous microRNAs and long non-coding RNAs such as maternally expressed gene 3 (*MEG3*) (5). These epigenetic processes are responsible for the spatial and temporal expression pattern of DLK1 throughout development and life.

We have previously reviewed the expression profile of DLK1 by tissue type in detail (6). DLK1 is expressed in many tissues during embryogenesis and somatic tissue development. After birth, however, DLK1 expression is silenced in most healthy tissues and is restricted to populations of stem/progenitor cells, most notably in endocrine organs (adrenal cortex, gonads, prostate) and the liver, where it is a marker of hepatoblasts (6).

DLK1 plays an important functional role in regulating the processes of tissue differentiation and development. This appears to be most crucial in embryonic development especially in skeletal muscle and adipose tissue. The burden of evidence suggests DLK1 has an important role in the inhibition of differentiation, which correlates with its expression data in adult tissues (6). The effect of DLK1 dose manipulation results in different effects on proliferation in different tissues. DLK1 knock-out (KO) mice are viable but present with retarded growth, skeletal malformation, obesity, and increased serum lipid metabolites (7). In adipose tissue, DLK1 KO appears to lead to earlier terminal differentiation and increased adipose proliferation. In muscle, DLK1 KO prevents muscle proliferation and DLK1 dose increase, as is seen in Callipyge sheep, leading to skeletal muscle hypertrophy from a young age. These sheep have a point mutation in the *DLK1/MEG3* intergenic region leading to increased expression of DLK1 and other genes from the paternal allele (8). Mice generated with double or triple doses of DLK1 exhibit *in utero* growth advantages; however, they present with failure to thrive and have associated increased embryonic mortality (9).

A similar picture is seen in humans, in genetic conditions where DLK1 is downregulated. Temple syndrome is caused by uniparental disomy of chromosome 14q32, leading to sufferers inheriting two imprinted copies of DLK1 (and other paternally expressed genes) and a double dose of the maternally expressed genes at this locus. Like the DLK1 KO mice, it is associated with growth retardation, lower birth weight, and increased incidence of diabetes mellitus and obesity. It also presents with precocious puberty, presumably due to earlier terminal differentiation of hypothalamic and pituitary progenitor cells (5). Precocious puberty is also attributed to separate genetic point mutations in DLK1, leading to loss of function, where patients present with a similar phenotype to Temple syndrome, highlighting the importance of loss of DLK1 driving the phenotypic changes rather than other perturbed genes at this locus (10). This highlights the physiological importance of DLK1 in different tissues to regulate the fine balance between differentiation and proliferation required for normal development.

There is not yet a consensus on how DLK1 exerts its functional effects. We have provided a detailed review of the collated evidence previously (6). Given the structural homology of DLK1 to canonical Notch ligand delta-like ligand 1 (DLL1), the mechanistic focus on how DLK1 exerts its function has been on Notch signalling. Collated evidence shows DLK1 to activate, inactivate, or not affect Notch signalling in different models. The evidence for the effect of DLK1 on Notch signalling is predominantly through differential expression of Notch target genes. Direct interaction between DLK1 and Notch 1 has been shown previously using two-hybrid assays (11). However, a recent paper has intriguingly thrown the mechanism of action of DLK1, specifically through direct Notch interaction, into question (12). They have identified that DLK1 binds to canonical TGF-β ligand receptor, activin receptor type 2B (ACVR2B). This was identified through proteomic data and confirmed through crystal structure analysis and functional experiments in human osteosarcoma (U20S) and murine myoblast (C2C12) cell lines. DLK1 is revealed to antagonise the canonical signalling at this receptor. Furthermore, they report that DLK1 does not interact directly with Notch 1 but can influence Notch activity through the inhibition of SMAD2/3 co-localising with the Notch intracellular domain (NICD) as a sequela of binding ACVR2B. This is an enticing discovery and could help to reconcile some of the contradictory functional effects of DLK1. For example, non-canonical TGF-β signalling can affect ERK and MAPK signalling, which is reported to be affected by DLK1 in other models (13, 14, 15, 16). However, there is not yet enough evidence to definitively discount DLK1 exerting some of its effect through Notch signalling in different models or through additional tissue-specific interacting proteins. Furthermore, existing evidence attributes some of DLK1’s function to nuclear translocation of the short intracellular portion of the full-length isoform after cleavage, which cannot be reconciled with this new interaction (16, 17). It seems likely that there are several mechanisms through which DLK1 exerts its function.

### DLK1 expression in cancer

Despite being absent from most healthy tissues, DLK1 is re-expressed at a high frequency across a wide range of different cancers. We have reviewed this in detail previously (6). The highest frequency of DLK1 expression is seen in endocrine and neuroendocrine tumours. It is a ubiquitous feature of adrenocortical carcinoma (ACC) (18, 19), paraganglioma, and phaeochromocytoma (20, 21). In addition, high expression is seen in medullary thyroid carcinoma (reported frequency 45–93%) (20, 22), ovarian carcinoma (67%) (23), small cell lung cancer (21–75%) (24, 25, 26), and lung carcinoid (83%) (26). DLK1 expression is also a feature of the childhood tumour neuroblastoma, although the frequency is not reported (27, 28).

Similarly, DLK1 expression is a ubiquitous feature of another childhood tumour, hepatoblastoma, and is also observed in hepatocellular carcinoma (HCC) (4–73%) (25, 29, 30, 31) and hepatocellular–cholangiocarcinoma (37–44%) (31, 32). Other common malignancies with reported DLK1 expression include breast cancer (39%) (25), non-small cell lung cancer (9–75%) (25, 33, 34, 35), and colon adenocarcinoma (59%) (25). Furthermore, it is reported in mesenchymal tumours, such as rhabdomyosarcoma (76%) (36) and gastrointestinal stromal tumours (GIST) (37%) (37), and is upregulated in haematological malignancies, particularly acute myeloid leukaemia (AML) (80%) (38).

In many of these cases, DLK1 expression is not only a feature of the malignancy but also appears oncogenic and is associated with worse prognoses. This is best described in endocrine-related cancers. In ACC, we showed that DLK1 expression is independently associated with worse recurrence-free survival (RFS) (19). Higher DLK1 expression is associated with worse overall and progression-free survival in high-grade serous ovarian carcinoma (23), neuroblastoma (28), and GIST (37). Worse RFS is also seen with higher DLK1 expression in HCC (25) and NSCLC (33, 35).

The re-expression of DLK1 in cancer not only represents a reversion to a de-differentiated or stem-like phenotype but also appears to have a genuine oncogenic role in driving malignancy. Like the lack of consensus on the biological mechanism of DLK1, it is unclear what the mechanism of DLK1 re-expression is in cancer. What is clear is that in almost all cases studied, this is not caused by biallelic expression and loss of imprinting (6). This is only reported as the mechanism for expression in a study in AML (38). Reported mechanisms for increased DLK1 expression include copy number alterations and epigenetic monoallelic mechanisms. To this end, a super-enhancer has been recognised at the DLK1 locus in 6/10 neuroblastoma cell lines, and this correlates with high DLK1 expression (27). In addition, DLK1 expression has been linked to increased Wnt/βCatenin activity in hepatoblastoma (39). In this model, DLK1 expression is driven through aberrant Wnt signalling through a Wnt-responsive element identified in the *DLK1/DIO3* locus. As with the mechanism, the stimulus for increase DLK1 expression is also unclear, although it has been shown to be in response to hypoxia in human neuroblastoma cells (17).

Although the exact mechanism of DLK1 re-expression in cancer and how it exerts its function are unclear, the wealth of evidence supports the fact that DLK1 potentiates malignancy. This is potentially a result of the inhibition of differentiation and acting as a pool of cancer stem or transiently amplifying cells to populate the cancer. In holding cells in a less differentiated state, there will be a natural resistance to common chemo and radiotherapeutics. In addition, in some models, DLK1 also drives proliferation and aggressiveness of disease (17, 36, 40). The oncogenic nature of DLK1, its preferential expression in cancer, absence in healthy tissues, and cell surface expression make it an ideal candidate for novel targeted therapies.

## Targeting DLK1

In the past 10 years or so, there has been a burgeoning interest in developing novel therapies to target DLK1. This has escalated in pace over the past 5 years, with several novel agents now in phase I trials. Although not all reported studies to date are focussed on targeting DLK1 in endocrine-related cancers, the results and strategies are especially relevant in drug development for these cancers, where DLK1 is expressed at the highest frequency. The different strategies in targeting DLK1 and a comprehensive review of all the results to date are detailed below.

### Monoclonal antibodies

Antibody-based therapies are an expanding field in oncology and precision medicine. The ability to generate monoclonal antibodies (MAbs) has been possible since the development of hybridoma technology in 1975 (41). Initially, these were mouse antibodies recognising a human target. This led to immunogenic reactions and reduced effector function of the antibody. Since then, development in recombinant DNA technology, genetic editing, and transgenic models has led to the ability to generate chimeric, humanised, and even fully human monoclonal antibodies. In addition, a separate strategy has been developed and refined, using phage display to generate fully human MAbs (42, 43).

The majority of therapeutic MAbs are generated as IgG1 subclass, due to preferential effector function and longer half-life of approximately 23 days in blood (44). The structure of IgG1 MAb is shown in Fig. 1B. Broadly, it consists of two heavy chains and two light chains to form the ‘Y’-shaped configuration. The top ‘arms’ of the Y are structurally identical fragment antigen-binding (Fab) regions, each containing a specific antibody-binding site. The ‘body’ of the Y is the fixed chain (Fc) region, which contains the interaction sites for ligands that are responsible for effector function, which includes three Fc receptor types (FcγRI, FcγRII, and FcγRIII) and the C1q complement component (45).

The primary effector function of therapeutic MAbs is antibody-dependent cellular cytotoxicity (ADCC). This effect is predominantly carried out through the recognition and binding of CD16+ natural killer (NK) cells to the FcγRIII receptor on the MAb, which has recognised and bound to the antigen on the cancer cell. Upon binding, the NK cell releases cytolytics and cytokines that enter the targeted cell causing apoptosis. This process is heavily dependent on the structure of an oligosaccharide in the fixed chain near the binding site of the NK cell (45). This is important because although some novel MAbs may have good intrinsic ADCC capabilities, binding at FcγRIII can be enhanced through structural manipulation of the oligosaccharide sequence. Specifically, this is the removal of a core fucose (afucosylation), and this is widely used to enhance ADCC activity. Importantly, afucosylation does not affect the activity of another effector function of MAbs, complement-dependent cytotoxicity (CDC) (45).

CDC occurs when C1q binds to the constant region of a cancer cell-bound MAb. This initiates a cascade of complement activation leading to the formation of a membrane–attack complex, causing cell penetration and cytolysis. Notably, the potency for this effect is more pronounced in the IgG3 subclass. This has been exploited to potentiate CDC by structurally engineering the IgG1 MAb to have a portion of the IgG3 fixed chain, termed ‘Complegent technology’. This has been shown to increase the effect of CDC and, importantly, does not affect the ADCC capability of the MAb (46).

To our knowledge, the only reported MAb targeting DLK1 through ADCC is from Chiome Bioscience in Japan. CBA-1205 is a novel afucosylated humanised MAb targeting DLK1 (47). They showed that CBA-1205 binds to human and cynomolgus monkey (but not rodent) DLK1 in the EGF1-2 region with high specificity. ADCC activity was confirmed in hepatic cancer cell models (Hep3B and Hep G2) *in vitro* both in co-culture with NK cells (TK176V) and in using human peripheral blood mononuclear cells as effector cells. Furthermore, it was effective in a dose-dependent manner at reducing tumour volume in two cancer xenograft models (Hep3B and Hep G2 injected into nude mice). It was also shown to have a synergistic effect with the tyrosine kinase inhibitor lenvatinib, which is used in hepatocellular carcinoma patients who are unable to receive immunotherapy. Importantly, toxicity assessed in cynomolgus monkeys did not reveal any serious adverse events with only mild liver inflammatory changes noted.

These results have led to a phase I trial in humans (NCT06636435). The first part of this trial was reported in early 2025 and showed that CBA-1205 was tolerated up to the maximum planned dose of 30 mg/kg with no serious adverse events (48). The study population was of 22 Japanese patients with a range of progressive malignancies (none overtly associated with DLK1 overexpression). Indeed, DLK1 positivity was unproven immunohistochemically in the 14 patients who had available primary tumour samples. Although this was primarily a dose escalation, safety, and tolerability study, there was reported disease stability and some tumour response. This occurred proportionately more in patients who had detectable DLK1 serum levels. One patient with melanoma showed a good response with stable disease through 34 cycles of treatment, having progressed through or not tolerated conventional melanoma immunotherapy. Future parts of this study will focus specifically on relapsed/refractory HCC patients, and we await the results with interest. There are no reported studies of any stage looking specifically at DLK1 MAbs in endocrine cancer, but these promising results give hope that similar methods can be exploited in these diseases. The therapeutic approach with most evidence to date in endocrine and neuroendocrine cancers is antibody drug conjugates.

### Antibody drug conjugates

In addition to MAbs targeting oncogenic neo-antigens through effector functions, another novel therapeutic approach is through antibody drug conjugates (ADC). ADCs are MAbs that have a cytotoxic payload attached by a linker to the Fc (Fig. 1C). The typical mechanism is one whereby the MAb will bind the target antigen, become internalised, and deliver the cytotoxic payload. Depending on the cleavage mechanics of the linker, there may also be cytotoxic delivery to adjacent cells. Despite the enticing prospect of this targeted therapy, those in trials and clinical practice often present with high levels of treatment-related adverse events (>90% in a recent meta-analysis of which >40% were grade 3) (49). Maximising drug delivery and minimising off-target toxicity involves optimisation of every aspect of the drug. This starts with the target that should be a cell surface protein, with preferential high-level expression in cancer but few healthy tissues. Theoretically, the protein target should be fixed and not cleavable to allow retention of the antigen and help minimise off-target effects. Next, the MAb needs to show specific binding leading to internalisation. As ADC technology has developed, biparatopic (targeting two separate epitopes of the same antigen) and biallelic (targeting two separate antigens) ADCs have been generated leading to more specific mechanism of action (50). The linker can be cleavable or fixed, and this will also affect the delivery of the payload.

Finally, there is a choice of payload. Newer generations of drugs use potent cytotoxic compounds that are effective at a much smaller dose than conventional chemotherapeutic agents. Currently, the commonly used payloads of current FDA-approved ADCs include monomethyl auristatin F (MMAE), monomethyl auristatin E (MMAF), maytansine derivatives DM1 and DM4, calicheamicins, topoisomerase I inhibitors, and pyrrolobenzodiazepine dimers (PBDs) (50). However, some of these payloads can be effluxed by ATP-binding cassette (ABC) proteins, and this needs to be borne in mind during drug design (51).

Currently, there are two ADCs targeting DLK1 in phase 1 trials. The first is called ADCT-701 and is a humanised IgG1 DLK1 MAb conjugated to PBD cytotoxin SG3199 via a cleavable linker (52). This was introduced on a poster at the meeting of the American Association of Cancer Research (AACR) in 2018 where it showed effective tumour control in xenograft mouse models of neuroblastoma and HCC. Subsequently, the same group has elaborately confirmed the candidacy of DLK1 as a target in neuroblastoma with a proteogenomic surfaceome approach and has presented more convincing data for the use of ADCT-701 in neuroblastoma (27). They showed that the MAb alone (without a conjugated payload) does not have any intrinsic anti-tumour efficacy. ADCT-701 was then tested in 13 xenograft neuroblastoma models with varying levels of DLK1 expression. Models with high DLK1 expression led to complete response, and this was even seen in models with heterogeneous DLK1 expression at presentation suggesting some bystander cytotoxic effect. These data have led to a phase I first-in-human DLK1-directed immunotherapy trial using ADCT-701 to treat neuroendocrine tumours, including neuroblastoma, in adult patients (NCT06041516).

The same agent, ADCT-701, has also been used in preclinical studies of other malignancies. Work from our laboratory has previously shown the ubiquitous expression and negative prognostic impact of DLK1 in ACC (18, 19). A recent study from the National Institute of Health in the United States has confirmed these expression findings in a smaller cohort and identified similar prognostic implications from the Cancer Genome Altas Pan-Cancer analysis project where 11/12 treatment-refractory ACC showed high expression of *DLK1* (21, 53). Using ACC cell lines *in vitro*, ADCT-701 was shown to have good cytotoxic effects on cell lines with good DLK1 surface expression, CU-ACC1, and H295R, but not in CU-ACC2, which has less surface expression. Interestingly, this occurred through both direct effects and bystander killing of DLK1-negative cells. These findings were not completely recapitulated in patient-derived organoid and patient-derived xenotransplant models, where acquired and intrinsic resistance to ADCT-701 was demonstrated in DLK1-positive tumours. This was revealed to be because of DLK1-regulated co-expression of drug efflux pump ABCB1 in DLK1-positive cells, leading to efflux of PBD. This appears to be a cancer-specific issue with DLK1-positive cells in ACC, as ADCT-701 was shown to provide a complete and durable response in SCLC tumours that lack ABCB1 expression. This study highlights the importance of payload choice in ADC design and how this must be considered within the cellular landscape of the targeted malignancy. It also proposes a possible explanation for the negative prognostic effect of DLK1; two of the main chemotherapeutic drugs in ACC (doxorubicin and etoposide) are known to be effluxed by ABCB1 (21).

ADCT-701 has also been used in preclinical testing of Down syndrome related to childhood myeloid leukaemia (ML-DS). A preprint from a study has shown the efficacy of ADCT-701 in treating ML-DS in patient-derived xenograft models (54). They also have shown that DLK1 expression is unique to leukaemic rather than adult haematopoietic cells in this model and that DLK1 expression is driven through an enhancer in the *DLK-DIO3* locus being occupied by the truncated form of GATA1 (GATA1s), which is a recognised molecular aberration of ML-DS (55).

Another ADC from a different group targeting DLK1 is called TORL-4-500. This is a humanised IgG1 MAb selective for DLK1 linked to monomethyl auristatin E (MMAE) by a cleavable linker. Preclinical studies presented at AACR in 2024 showed significant tumour regression in four DLK1-expressing human cancer cell line xenotransplantation models (HCC, 2 x SCLC, and sarcoma) but not in DLK1 non-expressing models. Like ADCT-701, the MAb shows high binding affinity and internalisation to human and monkey DLK1. Toxokinetics from monkey studies support safe rollout to humans and a phase I trial is ongoing (NCT06005740) (56, 57).

The collated data from the ADC agents targeting DLK1 are promising in the preclinical setting. Given the specificity of the MAb in these compounds to only human and monkey DLK1 and the predominant use of mouse xenotransplant studies, the full toxicity profile of these agents is not yet known. The phase I trials involving both drugs will be informative in this regard. The data also highlight the importance of the malignancy in drug design and how the future of effective ADC therapy will be in more tailored, cancer-specific approaches. At the time of writing, phase I ADC trials are still recruiting and no results have been published.

### Dendritic cell vaccines

Another therapeutic approach has focussed on targeting DLK1 in tumour-associated vascular pericytes. DLK1 expression is increased in vascular pericytes associated with murine cancers of the kidney, colon, and melanoma compared to normal tissue in the same animals (58). It is well established that a feature of cancer is disorganised blood supply. After a primary tumour grows larger than 1–2 mm^3^, an angiogenic switch occurs, partly driven by tissue hypoxia, leading to angiogenesis and vasculogenesis (59). Within the tumour architecture, these vessels are phenotypically distinct from normal and bear tumour blood vessel antigens (TBVA). Targeting oncogenic angiogenesis is an established treatment strategy, with antibodies to vascular endothelial growth factor (VEGF) and other anti-angiogenic drugs already in clinical use. Indeed bevacizumab, a VEGF MAb, has shown a clinically meaningful survival benefit in metastatic colorectal cancer (60).

A theoretical sustained anti-angiogenic response might be provided with an immune activation strategy against TBVAs. One such approach is using condition dendritic cells (DC). DC are crucial in the innate and adaptive immune system, presenting antigens to T-cells to initiate and maintain targeted immune response (61). It is possible to generate a vaccine using type 1 dendritic cells that have been conditioned against specific antigens so that, on infusion a CD8+ T-cell response occurs against the antigen. In this case, DLK1 as a recognised TBVA in the models above, has been used as the target antigen. A vaccine, raised in interleukin 12–modified DC pulsed with major histocompatibility complex class I–presented DLK1, was able to decrease tumour growth, increase apoptosis and inflammatory tumour cell infiltration in a mouse model of renal cell carcinoma (58). Work from the same group has subsequently shown in the same model that DC targeting of DLK1 leads to increased expression of DLK1 homologue and DLK2, and vice versa. Better anti-oncogenic effects are achieved by targeting both simultaneously (62). This approach leads to a normalisation of the vasculature with a theoretical improvement of physiological immune response. Chronic inflammation in the tumour microenvironment (TME) can lead to expression of immune checkpoint molecules such as programmed death (PD)-1, and this can be abrogated with anti-PD-ligand 1 (PD-L1) therapy in conjunction with the DLK1/2 DC vaccines. PD-L1 monotherapy did not lead to tumour benefit in the absence of the vaccines (62).

A similar experimental strategy has been used by a different group in a mouse model of colorectal carcinoma (CRC) (63). Type 1 DC conditioned against DLK1 were administered to mice carrying subcutaneous tumours from a murine CRC cell line (MC38). As above, this was given with PD-L1 blockade to prevent TME-instigated immunosuppression. Treatment led to tumour shrinkage, normalisation of the tumoural vasculature, and increased CD8+ T-cell infiltration in the tumour.

A phase II trial using this methodology has shown good safety and some efficacy in patients with immune checkpoint blockade-resistant melanoma (64). Monocyte-derived type 1 DC were conditioned with 6 TBVA including DLK1 and administered with the tyrosine kinase inhibitor dasatinib, which has previously been shown to enhance the efficacy of DC vaccine in a mouse model of melanoma (65). Objective clinical responses were achieved in six of the patients. All six patients exhibited specific CD8+ T-cell response to the vaccine-presented targets. Although the exact effect of DLK1 cannot be stripped out from this study, the only antigen to which all six patients developed a T-cell response to was DLK1.

Overall, the evidence to date suggests that DC vaccines targeting vascular pericyte DLK1 expression has promise. The safety data thus far from the only human phase II trial are good with remarkable treatment responses seen in some patients. This modality, however, does require other immune modulation, be it through PD-L1 blockade or with adjunctive other anti-oncogenic drugs. It is noteworthy that these studies have focussed on targeting DLK1 expression in the associated TME and vasculature of cancers rather than targeting DLK1 as a neo-antigen in the primary tumour cells, which is another established use of DC vaccines in cancer.

Although DC vaccines targeting DLK1 have not yet been tested in endocrine cancers, the results to date in other models offer promise that this strategy may be used in such disease. DC vaccines (with other targets) have been used in endocrine cancers that express high levels of DLK1, which is not only informative as a proof of concept but also highlights some specific considerations. Within this context, there are most data for the use of DC vaccines in ovarian carcinoma. Although there are few striking successes reported, the SOV01 trial for patients with epithelial ovarian carcinoma reported some promising and relevant results (66). In this trial, patients were treated with autologous DC that had been pulsed with two allogenic ovarian carcinoma cell lines. There was a significant increase in PFS in those undergoing DC vaccination following chemotherapy and subsequent analysis found that this effect was most pronounced in those with immunologically ‘cold’ tumours with lower tumour mutational burden (TMB) (67).

These findings are potentially of interest for other immunologically ‘cold’ endocrine cancers such as paraganglioma and phaeochromocytoma. Reported DC vaccination in these tumours is restricted to murine models; however, one study did show that chromogranin A can be effectively used as a specific target molecule for DC vaccination (68). Given the paucity of tumour-associated neo-antigens in such cancers with low TMB, targeting DLK1 in a DC vaccine, potentially using some of the existing approaches in trials above, offers potential. There are no reported data on the expression of DLK1 in the TME vasculature in these endocrine cancers; however, there is a theoretical promise of a direct antitumoural effect.

### Chimeric antigen receptor T-cell therapy

An alternative methodology for triggering a lasting intrinsic immune response to an oncogenic antigen is chimeric antigen receptor T-cell (CAR-T) therapy. This revolutionary therapy has changed practice in haematological oncology, especially in B-cell malignancies and myeloma. The principle is to genetically engineer patients’ own T-cells with a novel chimeric receptor to identify a cancer-specific antigen and generate a lasting immune response. The CAR itself consists of an antigen-recognising ectodomain, a hinge, a transmembrane region, and an intracellular signalling endodomain (Fig. 1D). The antigen-recognising domain is often generated from the variable region of MAbs and is linked together as a single chain of variable fragments containing very light and heavy chains of Ig. The endodomain of the CAR contains receptors of a variety of co-stimulatory proteins, which enhance proliferation and cytotoxicity (69). To date, there are no approved CAR-T-cell therapies available for solid tumours.

The only published report of DLK1-directed CAR-T-cell therapy is in HCC. A group in China has generated CAR-T-cells against human DLK1 and used them to treat human HCC cancer cell line (HepG2) tumours in nude mice (70). The treatment showed a remarkable reduction in tumour weight and size compared to a vehicle arm, which was the same CAR-T product recognising CD19. What is more, there were persistent human CAR-T-cells present in the mice treated with the DLK1 product 4 weeks after inoculation, which is consistent with the expected proliferation observed from contact with the target antigen. Again, a true study of off-target effects is not possible in this model as the CAR vector does not recognise mouse DLK1; however, this study is the first use of DLK1-directed CAR-T-cell therapy with results suggesting there is enormous promise.

It is enticing to speculate about the translatability of this approach when applied to endocrine cancers with preferential DLK1 expression levels. CAR-T–directed therapy has been reported in ACC recently and does highlight some important considerations. A group from Germany has generated CAR-T-cells targeting the oncofetal antigen receptor tyrosine kinase-like orphan receptor 1 (ROR1) (71, 72). The efficacy of this treatment in preclinical models is impeded by glucocorticoid expression from the ACC. It is well established that steroidogenic ACC carries a worse prognosis, and it is felt that this effect is in part due to an immunosuppressive effect of steroid excess, both overt and intratumoural. This group has edited the T-cell construct to delete the human glucocorticoid receptor leading to complete remission of steroid-producing ACC xenografts *in vivo*. This highlights an important consideration in the development of targeted immunotherapy in steroidogenic cancers.

The first in human trial testing ROR-1 CAR-T-cells is currently recruiting patients with ACC, ovarian cancer, mantle cell lymphoma, breast cancer, and chronic lymphocytic leukaemia (EUCT: 2024-512019-36-00).

### Radioimmunotherapy

Finally, DLK1 has also been investigated as a target of radioimmunotherapy. This technique involves the linkage of a radionuclide to an MAb that is then incorporated into tumour cells leading to cell death. There is increasing interest in using alpha emitting radionuclides in this context, particularly Astatine 211 (73). In a study in lung cancer and neuroblastoma, a Japanese group had shown a proof of concept whereby DLK1 MAb conjugated to Iodine 125 (as a halogen surrogate for the alpha-emitting Astatine 211) is taken up in both SCLC (Lu-135) and neuroblastoma (SK-N-F1) cell lines *in vitro* (33). This was further assessed in mice injected with SK-N-F1 cells where radioactive antibody was taken up into tumour cells; however, there was also significant take up in other healthy tissue sites, most notably blood.

## Conclusion

The importance of DLK1 re-expression in cancer is becoming increasingly well established. It is a ubiquitous feature in certain endocrine and neuroendocrine tumours and is frequently observed in many other common malignancies as described. DLK1 expression carries negative prognostic outcomes in the cancers in which this has been studied. DLK1 clearly marks populations of progenitor/stem cells within the cancer. Whether this drives malignancy through potentiating metastatic spread or by conferring intrinsic resistance to common treatment strategies is unclear. These data, along with other preclinical studies of oncogenic behaviour, have established the candidacy of DLK1 as a target in novel cancer therapies.

Although the reported data of targeting DLK1 in endocrine cancer thus far are limited to ACC and neuroblastoma, approaches in the other malignancies as described are enormously relevant. As a class, endocrine and neuroendocrine malignancies have the highest frequency of DLK1 expression across cancer, and there is no evidence that DLK1 differs as a protein target in other more common, non-endocrine malignancies that have been studied above.

The candidacy of DLK1 as a target across the breadth of cancer is well established. Modern strategies for targeting cancer neo-antigens rely on abundant cell surface expression in cancer cells and low expression in health tissues. DLK1 fulfils these characteristics perfectly. There is a theoretical benefit to not targeting a cleavable cell surface protein as this may prevent internalisation and may lead to off-target effects. It is reassuring, therefore, that in the studies presented above, across the different modalities, there is evidence of targeted engagement with cell surface DLK1 leading to the intended cytotoxic effects. Whether this is mediated through interaction with the membrane-bound isoform of DLK1 or altered cleavage conditions of the full-length ligand, it is not yet known. Given the success of these novel strategies in targeting tumoural DLK1-expressing cells, it is essential that further studies assess for off-target effects. Progress in phase I studies of DLK1 targeting agents in other (non-endocrine) cancer settings is valuable in the design of novel agents regardless of the target malignancy. The published data from MAb therapy (CBA-1205) suggest that there are few observed issues (47, 48). Notably, there was a mild hepatic inflammatory change observed in cynomolgus monkeys, and no serious adverse events were noted in the only reported phase I trial in humans. The outcomes of the two phase I trials of DLK1 ADCs (ADCT-701, TORL-4500) will be informative as this therapy is potentially more toxic than MAbs alone.

The off-target effects of DLK1-targeted agents will be relevant in the drug development and translation of these existing agents to endocrine and neuroendocrine cancers. An additional consideration is how innate features of these malignancies might affect the on-target effects, in particular, considering ACC and phaeochromocytoma/paraganglioma as the malignancies where DLK1 expression is ubiquitous. Both tumour types are relatively immunologically ‘cold’, and in ACC this is associated with increased steroidogenesis (52). Therefore, the results of the SOV01 trail in epithelial ovarian carcinoma are important, in which dendritic cell vaccines were more efficacious in the immunologically ‘cold’ tumours (66). Dendritic cell vaccines have shown slower progress in drug discovery than other novel methods mentioned above, which, in part, is due to a lack of suitable tumour-specific antigens to target. Although not yet studied as a DC vaccine target for primary tumour cells in any cancer, a DLK1-targeted DC vaccine approach may prove a novel option in phaeochromocytoma/paraganglioma and other such immunologically ‘cold’ tumours with low TMB. In ACC, however, immune targeting is restricted by steroidogenesis. The more novel approach with CAR-T-cell therapy, especially where the glucocorticoid receptor has been deleted, provides enormous hypothetical potential with promising *in vitro* data (71, 72). This is particularly fascinating as it may highlight the use of ACC as a model for improving CAR-T-cell delivery in other non-steroidogenic cancers.

The alternative in ACC is a strategy using MAbs, especially in the form of ADC. It is theoretically possible to engineer a DLK1 MAb ADC construct that has been afucosylated to enhance ADCC, has a chimeric IgG1/3 Fc to enhance CDC, and has a cytotoxic payload that is not effluxed by ABCB1 or other known drug efflux pathways. This would offer a multimodal approach to a DLK1-targeted treatment in this malignancy, which could offer wide translatability to other endocrine and more common DLK1-expressing cancers.

Overall, it is clear to see why there is an exponential growth of interest and research into targeting DLK1 in cancer. With one phase II and three phase I trials ongoing, it feels like the approval of a DLK1-targeted therapy in endocrine cancer cannot be far away.

## Declaration of interest

The author declares that there is no conflict of interest that could be perceived as prejudicing the impartiality of the research reported.

## Funding

This work was supported by a Clinical Lecturer Starter Grant from the Academy of Medical Sciences (SGCL033\1077), an NIHR Academic Clinical Lectureship (CL-2024-19-003), and a Translational Research Grant from the Nefkens Adrenal Cancer Foundation (2025013 DLK1-Guasti).
